# Flexible ureterorenoscopy is associated with less stone recurrence rates over Shockwave lithotripsy in the management of 10-20 millimeter lower pole renal stone: medium follow-up results

**DOI:** 10.1590/S1677-5538.IBJU.2017.0483

**Published:** 2018

**Authors:** Faruk Ozgor, Murat Sahan, Fatih Yanaral, Metin Savun, Omer Sarilar

**Affiliations:** 1 Department of Urology, Haseki Teaching and Research Hospital, Istanbul, Turkey

**Keywords:** Calculi, Lithotripsy, Recurrence

## Abstract

**Purpose:**

To identify the role of shock wave lithotripsy (SWL) and flexible ureterorenoscopy (f-URS) on the stone recurrence, in the management of 10-20 millimeter lower pole stone (LPS) with medium follow-up outcomes.

**Materials and Methods:**

The patients’ charts which were treated with SWL or f-URS for LPS between January 2011 and September 2013 were analyzed, retrospectively. Patients who had a solitary 10-20mm LPS were enrolled into the study. In both procedures, patient was accepted as stone free, if complete stone clearance was achieved in the 3rd month abdominal computed tomography. Only patients with a stone free status were evaluated in follow ups.

**Results:**

The stone-free rate was 77.9% (88/113 patients) for the SWL group and 89% (114/128 patients) for the f-URS group (p=0.029). Stone recurrence was detected in 28 (35.4%) patients in SWL group and in 17 (17.2%) patients in f-URS group (p=0.009). Stone types and 24 hour urine sample results were similar between groups (p=0.123 vs p=0.197, respectively). Multivariate regression analysis revealed that f-URS procedure and absence of abnormality in 24 hour urine analysis significantly decreased stone recurrence in medium term follow-up (p=0.001 and p<0.001, respectively).

**Conclusions:**

Our study showed for the first time, that patients which underwent f-URS for LPS, faced less stone recurrence, independent from diet regimen and metabolic evaluation in medium term follow-up. Additionally, presence of abnormality in 24 hour urine analysis increase the stone recurrence risk in follow-ups.

## INTRODUCTION

Management of lower pole stones (LPS) is still a challenging issue for urologists. Arguments focusing on the best treatment option for LPS are mostly on stone size and lower pole anatomical characteristics including infundibulopelvic angle, calyx length and calyx width (1). Open renal stone surgery has been abandoned in recent years, and indicated in only specific conditions. On the other hand, percutaneous nephrolithotomy (PNL) provides excellent stone free rates for LPS regardless of pelvicaliceal anatomy and stone size, but the procedure itself has some potential serious complications (2). Thus, flexible ureterorenoscopy (f--URS) and shock wave lithotripsy (SWL) gained popularity all over the World, especially in smalland medium-sized LPS.

Studies that investigated the effectiveness of SWL and f-URS mostly concluded that f-URS had higher stone free rates than SWL (3, 4). However, SWL has advantages such as being an outpatient procedure, with minimal anesthesia requirement and better patient acceptance. Conversely, flexible ureterorenoscopes may reach the entire pelvicaliecal system, including the lower pole and holmium laser provides effective stone fragmentation regardless of stone composition. Additionally, repositioning of a lower calyx stone to a more appropriate calyx facilitates stone fragmentation. Also, use of an ureteral access sheath provides lower intrarenal pressure, lower infection rate and facilitates the passage of stone fragments, which improves patients quality of life after the procedure (5).

Although many studies have compared the effectiveness of SWL and f-URS in the management of LPS, no studies in the literature have investigated the medium term follow-up results of these procedures. In this study, we aimed to compare the role of SWL and f-URS on stone recurrence, in the management of 10-20 millimeter LPS with medium term follow-up results.

## MATERIAL AND METHODS

In a tertiary academic center, the charts of patients’ who were treated with SWL or f-URS for LPS between January 2011 and September 2013 were retrospectively analyzed. Patients who had a solitary 10-20mm LPS were enrolled in the study. The medium term follow-up was defined as outcomes between 24 and 60 months after operation (6). The exclusion criteria were patients aged <18 years, patients with renal abnormalities, patients with concomitant ureteral stone, and patients in which ureteral access sheath could not be inserted. Patients with <24 months follow up were also excluded from the study.

In all patients, medical history was obtained and a detailed physical examination was performed. Stone size and calyx anatomy of the lower pole were assessed using intravenous pyelography or/and computerized tomography (CT) with urogram before the procedure. The estimated glomerular filtration rate (eGFR) was calculated in accordance with ‘Modification of Diet in Renal Disease Study’ equation (7). The selection of procedural technique was primarily based on the patients’ choice. Before each procedure, a sterile urine culture was obtained from every patient. All patients signed an informed consent form before undergoing SWL or f-URS.

### SWL Technique

A standard SWL was performed as an outpatient procedure under sedation analgesia. We did not insert JJ stent previous to the SWL in this study sample. We used a Dornier Compact Sigma (Dornier MedTech GmbH, Wessling, Germany) for SWL procedures. A total of 2000-2500 shocks were delivered per session (energy level 1-4, frequency 60-90 Hz). The procedure was started with a frequency of 60 shocks/min and energy level of 1, then the energy level was increased up to a level of 4. Frequency was increased to a maximum of 90 according to patient tolerance. Patients were evaluated with a Kidney Ureter Bladder (KUB) X--ray 1 week after each SWL session. The SWL sessions were given to a maximum of 3 sessions. The duration of the procedure was calculated as the sum of SWL sessions.

### f-URS Technique

Under general anesthesia, a safety guide--wire was placed into the pelvicaliceal system and semi-rigid ureteroscopy was used for visual evaluation of the ureter and to facilitate insertion of a ureteral access sheath (9.5/11.5Fr or 11/13F). A 7.5F fiber-optic flexible ureterorenoscope (Storz FLEX-X 2, Tuttlingen, Germany) with a 200 or 273μ m laser fiber (Sphinx Holmium Laser, Katlenburg/Lindua, Germany) was used for stone fragmentation, with an energy of 0.8–1.5 J and a rate of 5–10 Hz. Retrieval with a basket was preferred for stone fragments >2 mm and stone fragments <2 mm were left for spontaneous passage. At the end of each procedure, we routinely evaluate the ureter with semirigid ureterorenospy for any ureter injuries and a 4.8F JJ stent was placed. The operation time was accepted as the time passed from anesthesia induction to the placement of JJ stent. In the 2^nd^ week after operation, JJ catheter was removed using a cystoscope.

In both procedures, patients were considered stone free, if complete stone clearance was achieved in the 3^rd^ month abdominal CT. Complications were classified in accordance with the Clavien system (8). For each patient, the impact of diet regimen on stone recurrence was explained and high fluid intake with a protein- and salt--restricted diet was suggested. The diet regimen was accepted as successfully accomplished if the patients’ fluid intake was over 2500cc, salt intake was less than 2300 mg and meat intake was less than 6 ounces per day.

Patients who required a different procedure due to their residual stones during the follow-up period, were excluded from medium-term follow--up evaluation because of the potential effect of the other procedures on success and recurrence rates. Only patients with a stone free status were compared between groups in follow-ups; these patients were evaluated twice per year through personal interviews, a serum biochemistry panel, a 24-hour urine study, and kidney imaging (including KUB and abdominal ultrasonography). Abdominal CT was performed annually. Moreover, we recommended stone analysis for each patient. If any metabolic disorder was identified, medical prophylaxis was given. Recurrence was defined as new stone occurrence.

During statistical analysis values were evaluated as numbers, means, percentages and intervals. Numbers and percentages were compared using the Chi-square test. Before the comparison of means of values, the values were evaluated for homogenity. Homogeneously distributed values were compared using Student's t-test and heterogeneously distributed values were compared using the Mann Whitney U test. Multivariate regression analysis was used to identify importance of operation type and metabolic evaluation on stone recurrence. Statistical significance was considered when a p value was <0.05.

## RESULTS

One hundred thirteen patients and 128 patients were treated with SWL and f-URS, respectively. Patients preoperative characteristics were comparable between the groups in terms of age, ASA score, body mass index (BMI) and stone opacity (p=0.158, p= 0.128, p=0.293 and p=0.121, respectively). Also, stone size and eGFR values were similar between groups (p=0.143 and p=0.324, respectively). Preoperative parameters are summarized in Table-1.

**Table 1:** Comparison of preoperative demographics of patients.

		Groups		
		f-URS	SWL	p value
Number		128	113	
Gender (Male/Female)		63/65	65/48	0.246
Age* (years)		45.9±14.7	48.6±14.9	0.158
BMI* (kg/m^2^)		26.3±5.1	25.6±5.2	0.293
ASA Score		1.09±0.71	1.21±0.47	0.128
Stone size* (mm)		12.1±5.0	11.3±3.1	0.143
Operation side (R/L)		75/53	60/53	0.466
Stone opacity (opaque/non-opaque)		117/11	95/18	0.121
Initial GFR *		91.4±28.6	94.8±24.3	0.324
**Chronic Kidney Disease Classification**				0.126
	CKD 1	69	68	
	CKD 2	42	36	
	CKD 3	17	7	
	CKD 4	0	2	
	CKD 5	0	0	

* = Mean

**BMI** = Body mass index; ASA score = American Society of Anaesthesiologists Score

The mean operation time for SWL and f--URS groups was 25.9±2.5 and 52.3±16.2 minutes (min), respectively (p<0.001). The number of sessions per patient was significantly higher in SWL group (2.8 vs. 1.05, p<0.001). However, the mean hospitalization time was significantly longer in the f-URS group (p<0.001) (Table-2).

**Table 2:** Comparison of perioperative parameters and outcomes.

		Groups		
		f-URS	SWL	p value
Number		128	113	
Operation time (minutes)*		52.3±16.2	25.9±2.5	< 0.001
Hospitalization time (hours)*		21.1±16.6	2.18±0.87	< 0.001
**Complications**				0.181
	Grade 1	12	15	
	Grade 2	5	1	
	Grade 3a	2	0	
	Grade 3b	1	1	
Number of session		1.05±0.22	2.8±1.1	<0.001
Stone free status		114 (89%)	88 (77.9%)	0.029

* = Mean

Post-operative complications were similar between the groups (p=0.181). Transient hematuria (9 patients in SWL group and 10 patients in f-URS group) and renal colic (6 patients in SWL group and 2 patients in f-URS group) were the most common complications. Post-operative fever that required antibiotic therapy was observed in one patient in the SWL group and five patients in the f-URS group (Clavien grade 2). A JJ stent was re-inserted under local anesthesia for 2 patients in the f-URS group due to stent migration after the procedure (Clavien grade 3a). Semi rigid ureteroscopy was performed under general anesthesia for one patient in each group due to *strainstrasse* (Clavien grade 3b). The stone-free rate was 77.9% (88 patients) for the SWL group and 89% (114 patients) for the f-URS group and significantly higher in the f-URS group than in the SWL group (p=0.029) (Table-2).

The mean follow-up period was 32.4 and 34.1 months in the SWL and f-URS groups, respectively (p=0.242). In total, 24 patients (9 in SWL group and 15 in f-URS group) were lost to follow-up. The final eGFR was similar between groups (p=0.678). Also, the subgroups of chronic kidney disease (CKD) in each group were comparable (p=0.192). During follow-up, stone free status was observed in 82 of 99 patients (82.8%) in f-URS group and 51 of 79 patients (64.6%) in SWL group. Stone recurrence was more common in SWL group. Stone recurrence occurred in 28 patients in the SWL group and in 17 patients in the f-URS group (p=0.009). The recurrent stone size was 5.7 and 6.3 mm in f-URS and SWL groups, respectively (p=0.732). Furthermore, stone recurence period was comparable between groups (22.3 vs. 24.1, p=0.586) (Table-3).

**Table 3:** Comparison of follow up outcomes of patients.

		Groups		
		f-URS	SWL	p value
Number		114	88	
Follow-up time (months)*		34.1±13.2	32.4±8.5	0.242
Patients with follow up outcomes / Patients loss during follow-up		99/15	79/9	0.675
Patients compliance to diet regimen Yes/No		71/28	62/17	0.390
Patients use medical prophylaxis Yes/No		38/61	29/50	0.941
Stone Composition				0.123
	Calcium Oxalate monohidrate	44	29	
	Calcium Oxalate dihidrate	19	15	
	Calcium Phosphate	3	1	
	Magnesium ammonium phosphate	3	1	
	Uric Acide	9	15	
	Cysteine	5	0	
	No analyses	16	18	
**24 hours urine analysis**				0.197
	Normal	59	49	
	Hypocitraturia	12	11	
	Hyperoxaluria	6	3	
	Uricosuria	5	10	
	Cystinuria	5	0	
	Two or more abnormalities	7	4	
	No data	5	2	
Final GFR		95.2±32.8	93.6±26.1	0.678
**Chronic Kidney Classification**				**0.192**
	CKD 1	48	39	
	CKD 2	36	30	
	CKD 3	13	6	
	CKD 4	2	4	
	CKD 5	0	0	
Recurrent stone size* (mm)		5.7±2.4	6.3±2.6	0.732
Recurrence time		22.3±7.8	24.1±8.4	0.586
Final status	
Stone free/Stone recurrence		82/17	51/28	0.009

* = Mean

Compliance with diet regimen and the use of medical prophylaxis were not statistically significant between groups (p=0.390 vs. p=0.941, respectively). Also, results of 24-hour urine analysis were similar between groups (p=0.197). Hypocitraturia was the most common metabolic abnormality in both groups. Stone analysis was available in 61/79 and 83/99 patients in SWL group and f-URS groups, respectively. Calcium oxalate stones were the most common stone type in both groups and stone types did not show any significant difference between groups (p=0.123) (Table-3).

Comparison of patients with maintained stone free status and patients with stone recurrence in follow-ups, revealed that f-URS procedure and absence of abnormality in 24-hour urine analysis had a protective effect on stone recurrence (p=0.005 and p<0.001, respectively) (Table-4). Multivariate regression analysis showed that f--URS procedure decreased stone recurrence 5.5 fold and presence of abnormality in 24-hour urine analysis increased stone recurrence 29.9 fold, respectively (Table-5).

**Table 4:** Operation type and metabolic properties of patients according to stone free status in follow-up

	Groups		
	Stone free	Stone recurrence	p value
**Number**	133	45	
**Patients compliance to diet regimen Yes/No**	103/30	30/15	0.152
**Patients use medical prophylaxis Yes/No**	51/82	16/29	0.740
**24 hours urine analysis (Normal/Abnormal)**	107/26	8/37	<0.001
**f-URS / SWL**	82/51	17/28	0.005

**Table 5:** Multivariate regression analysis of patients according to stone free status in follow-up

	Odds Ratio*	p
Patients compliance to diet regimen	2.461 (0.911-6.647)	0.076
Patients use medical prophylaxis	1.677 (0.659-4.265)	0.278
24 hours urine analysis	29.920 (10.717-83.533)	<0.001
f-URS / SWL	5.580 (2.098-14.838)	0.001

Lojistic Regression Analysis

* = 95% confidence interval

## DISCUSSION

The therapeutic approach for LPS is varied, nevertheless, the best treatment modality is still under investigation. Patients with small LPS can be managed with observation but nearly 30% of these patients experience pain and an increase in stone size during follow-up. Moreover, spontaneous passage of LPS is difficult because of lower pole anatomic properties and gravity (9). For patients with LPS between 10-20mm and without unfavorable anatomic characteristics for SWL, the urology guidelines recommended SWL or endourologic procedures on equal terms (10). However, f-URS seems to be a less invasive procedure when compared with PNL.

SWL has been the most preferred treatment modality for LPS <2cm for a while. Gerber et al. stated that 65% of urologists preferred SWL for the treatment of intermediate size LPS (11). However, the efficacy of SWL is adversely affected by many factors such as obesity, longer skin-to-stone distance, and hard stones. Also, the success rates of SWL are lower in LPS compared with middle and upper calyceal stones. The stone free rate (SFR) for LPS following SWL was reported in a wide range from 44.6% to 90% (12). We achieved 77.9% SFR after SWL in the present study and our success rate was comparable with other studies in the literature.

Many authors demonstrated that f-URS provided significantly higher stone free rates in the management of LPS compared with SWL. EL--Nahas et al. reported 86.5% and 67.7% SFRs in the treatment of 10-20 mm LPS following f-URS and SWL, respectively (13). Singh et al. obtained 82.8% SFR after f-URS, for LPS in the same size (4). Similarly, SFR following f-URS was significantly higher in the present study. We explain these results in two ways. First, intracorporeal lithotripsy with laser fragmentation overcomes problems associated with higher BMI, longer skin-to-stone distance, and hard stones. Secondly, we relocated the LPS to a more appropriate location for fragmentation in 47.6% of cases and this may explain the higher SFR following f-URS.

The assessment modality for stone free status after SWL and f-URS were varied in different studies. Some authors used only KUB or only ultrasonography. Others preferred a combination of KUB and ultrasonography (3, 14). Pearle et al. preferred CT for the evaluation of success (15, 16). Studies have demonstrated higher efficiency of CT, thus we preferred CT to obtain more accurate results.

Recently, there is no data available on recurrence rates of LPS after f-URS in medium term follow up. Our study showed a significantly lower recurrence rate following f-URS in multivariate regression analysis (OR: 5.58, 95%, Cl: 2.09–14.83, p<0.001). We have two explanations to clarify that outcome. First, stones were fragmented using a Holmium laser and small fragments were extracted by basket during the f-URS procedure. On the other hand, to achieve stone clearance, spontaneous passage of fragments was expected after SWL sessions. These small fragments act as a nidus for stone growth and recurrence. Secondly, re-positioning of stones during f-URS procedure may have facilitated spontaneous passage of fragments, which is emphasized to be easier for fragments in the renal pelvis, upper and middle poles than in the lower pole. In SWL, fragmentation occurred in the lower pole and gravity prevents spontanous passage of fragments, which may be another reason of the higher recurrence rate after SWL.

The relationship between renal stone disease and deterioration of renal function is well known. Urinary tract infections, obstruction and frequent urologic interventions may have a role for renal function deterioration. Jungers et al. showed that nephrolithiasis related end-stage renal disease in patients was 3.2% (17). However, no patients in our study had CKD 5 renal disease and only four patients had CKD 4 renal disease in the SWL group. Our study sample may be a reason. Lower pole stone with 10-20 mm size rarely causes hydronephrosis or lead only to local calyceal ecstasy. The percentage of CKD subgroups was similar between the groups in the follow-ups.

Dietary regimens have a crucial role in stone recurrence. Dussol et al. reported a significant reduction in recurrence rates with diet regimen including low-animal-protein diet and a high-fiber diet (18). Patient's compliance to diet regimen was high and similar between the groups (p=0.390). However, we are aware that the compliance evaluation was only assessed using the patients subjective answers.

Previous studies demonstrated the importance of stone analysis and metabolic evaluation on stone recurrence. Altunrende et al. suggested that the presence of struvite stones was a risk factor for stone regrowth (6). Also, cystine and uric acid stones are associated with high recurrence rates. We were able to perform the stone analyses in 144 patients (83 patients in the f-URS group and 61 patients in the SWL group). The failure to achieve stone fragments during urination after SWL session resulted in lower patient numbers with stone analysis. Also, the Turkish healthcare system does not pay for infrared spectroscopy or X-ray differaction procedures. This fact may be another explanation why all patients did not have stone analysis. On the other hand, 94.9% of patients had 24-hour urine analysis and multivariate regression analysis revealed that presence of abnormality in 24-hour urine analysis increased stone recurrence 29.9 times fold.

Although this study is the first to investigate the effect of f-URS and SWL on 10-20 mm LPS with medium term results, our study had some limitations. First, the study had a retrospective design. However, all data were recorded prospectively in electronic database. Secondly, stone composition was not available in some patients, especially in the SWL group because of the possible reasons mentioned above. However, 94.9% of our patients had 24- hour urine samples to overcome this problem. Lastly, we did not analyze the effects of f--URS and SWL on patients’ quality of life and the economic burden to the health care system.

## CONCLUSIONS

Our study demonstrated that f-URS achieved better SFR in the management of 10-20 mm LPS over SWL. Moreover, we showed for the first time that patients who underwent f-URS faced less stone recurrence, independent from diet regimen and metabolic evaluation in medium term follow--up.

## ABBREVIATIONS

LPS: Lower pole stone
PNL: Percutaneous nephrolithotomy
f-URS: Flexible ureterorenoscopy
SWL: Shock wave lithotripsy
CT: Computerized tomography
eGFR: Estimated glomerular filtration rate
KUB: Kidney Ureter Bladder X-ray
BMI: Body mass index
SFR: Stone free rate
CKD: Chronic kidney disease
